# Science is perception: what can our sense of smell tell us about ourselves and the world around us?

**DOI:** 10.1098/rsta.2010.0117

**Published:** 2010-08-13

**Authors:** Jennifer C. Brookes

**Affiliations:** The London Centre for Nanotechnology, University College London, Gower Street, London WC1E 6BT, UK

**Keywords:** signal transduction, swipe card, olfaction, olfactory receptor, odorant, electron tunnelling

## Abstract

Human sensory processes are well understood: hearing, seeing, perhaps even tasting and touch—but we do not understand smell—the elusive sense. That is, for the others we know what stimuli causes what response, and why and how. These fundamental questions are not answered within the sphere of smell science; we do not know what it is about a molecule that … smells. I report, here, the status quo theories for olfaction, highlighting what we do not know, and explaining why dismissing the perception of the input as ‘too subjective’ acts as a roadblock not conducive to scientific inquiry. I outline the current and new theory that conjectures a mechanism for signal transduction based on quantum mechanical phenomena, dubbed the ‘swipe card’, which is perhaps controversial but feasible. I show that such lines of thinking may answer some questions, or at least pose the right questions. Most importantly, I draw links and comparisons as to how better understanding of how small (10’s of atoms) molecules can interact so specially with large (10 000’s of atoms) proteins in a way that is so integral to healthy living. Repercussions of this work are not just important in understanding a basic scientific tool used by us all, but often taken for granted, it is also a step closer to understanding generic mechanisms between drug and receptor, for example.

## 1. Introduction

Aristotle wrote in his major treatise, ‘On the Soul’, that ‘Generally, about all perception, we can say that a sense is what has the power of receiving into itself the sensible forms of things without the matter, in the way in which a piece of wax takes on the impress of a signet ring without the iron or gold’. To paraphrase: perception is the shadow, the imitator, the model. Perception is a tricky matter, and perhaps, at this moment, beyond the reaches of scientific rationale. However, to scrutinize Aristotle’s analogy of the impression by the ring on the wax (the initiation of a sensory process), the interaction humans have with the world at the first stages is very much *with* the matter. For instance, we absorb stimulating photons, packets (quanta) of light, that activate rods and cones in our eyes. We conduct the compressions and rarefactions of sound waves into our ears. We react with the acidic (−COOH carboxyl groups) on our tongue. In these first stages of recognition we interact with the world in a way that is much more intimate, though not to belittle the power and impressive nature of memory, than in the stages of perception and recall. It is imperative to realize that, in the first stages of any sensory process, we are *physically* interacting with the outside world.

**Figure 1.**
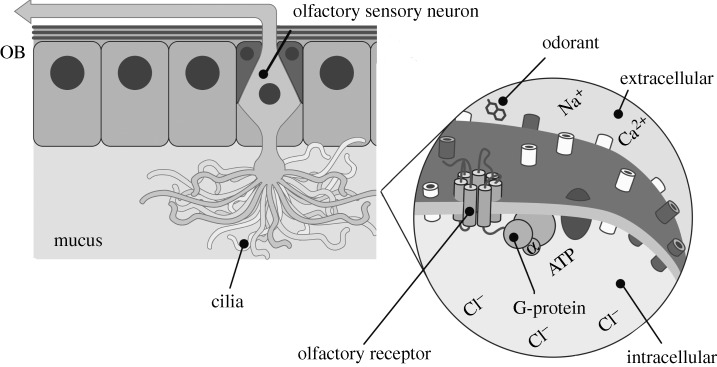
The olfactory epithelium where the odorant meets the central nervous system is shown. Olfactory receptors are the gate keepers that determine signal firing. They are found at the interface of the olfactory cilia and control whether or not an odorant will initiate a signal transduction process that results in the depolarization of the olfactory sensory neuron. The electric signal generated is projected onto the olfactory bulb (OB). Adapted from a presentation by Simon Gane.

Smell is arguably the most intimate of all the senses. When we smell an odorant molecule, it is volatile and non-reacting. These molecules are small enough to reach deep into the nose cavity, diffuse through a 10–40 μm thick mucus layer (Graziadei 1971), meet one of tens of thousands of cilia that project from the olfactory sensory neurons, and absorb onto one of the approximately 347 receptor types at the *extra*cellular–*intra*cellular interface (Buck 2004). There are thus approximately 347 related and various olfactory receptors that are presumably ‘tuned’ sometimes exclusively to, sometimes not, odorants (Malnic *et al.* 1999). Some receptors are broadly and some are selectively ‘tuned’. A whole lipid bilayer stabilizes and is responsible for the olfactory receptor proper orientation, each receptor has seven helices that cross the membrane. It is here where the initial interactions occur between the outside environment and our central nervous system that response receptors sit (figure 1). At recognition, the odorant is the first messenger, the receptors, *G-protein-coupled receptors* (GPCRS), release a G-protein unit and a second messenger process of transduction ensues that controls a Ca^2+^ and Na^+^ ion influx into the cell. Subsequent to this, Ca^2+^ ions act as third messengers that induce Cl^−^ ions to flow. This in turn causes the olfactory sensory neuron to fire. In this manner an odorant molecule message becomes an electric signal to be interpreted by the brain. Yet an often overlooked step is that the odorant receptor has to be ‘turned on’ to transmit this electricity, the intimate step being the gate keeper that determines recognition or ignorance, transmission of a signal or not. There is no obvious explanation as to why particular odorants open particular gateways. This is the most curious question in olfaction—‘What turns on the receptor?’—what is it about the matter, the odorant, that we are interacting with? What initiates transmission?

## 2. Past and present theories of olfaction

Aristotle’s figurative description has survived since 350 BC and even taken literal formation in the context of what is named the ‘lock and key’ description of olfaction first purported in 1963 by Amoore. The lock and key description states that to produce a particular scent a particular fit is required between the odorant and receptor (Amoore 1963). As in many enzyme recognition processes, well described for example by Sigala *et al.* (2008), the receptor recognizes an odorant via shape complementarity as the odorant ‘key’ fits the receptor ‘lock’.

This description, however useful elsewhere, does not work in olfaction. Different molecules that could fit the same site of a receptor more often than not do not smell the same (Sell 2006). Further, on the other hand, physiological studies of rodent olfactory receptor neurons have shown that olfactory cells respond to many odorants that are *not* the same shape (Tareilus *et al.* 1995; Rawson & Gomez 2002). Therefore, any predictions based on shape alone will give surprising results. Furthermore, the lock and key model does not explain what happens next in the process of smell. What does shape complementarity achieve? How mechanically can a small odorant (key) initiate global changes in a much larger and floppier^1^ protein (lock) when it is physically not comparable to an actual lock and key? Therefore, as a mechanism of signal initiation, lock and key alone cannot provide an explanation of signal *transduction*.

Mori & Shepard (1994) offer an alternative variation on the lock and key model: the ‘odotope theory’, in which key features (shapes) of the odorant are detected by the receptor rather than the shape as a whole. It may be one structural feature of a molecule, such as that of the functional group, that a particular receptor responds to as opposed to the general shape of that molecule. This theory placed the importance on the atoms present rather than on the position of the atoms. Further, this notion better represents the known ‘combinatorial code’ nature of odorant signalling (Malnic *et al.* 1999), whereby one odorant will activate several receptor types and one receptor type will respond to many odorants. One main objection to this model is the existence of the many well-documented cases of chiral molecules (handed, mirror image molecules or enantiomer pairs) that smell different in their mirror image forms. If the receptors detect individual groups contained on the molecule as opposed to the molecule as a whole, then the famous right-handed *4R*-(−)-carvone (‘spearmint, fresh herbal’) and left-handed *4*S-(+)-carvone (‘caraway, fresh herbal’) should smell the same. They, however, do not (Leffingwell and associates).

Quite different from any shape structure-based theory came the idea that a molecule’s vibrational spectrum determines its scent, purported by Dyson (1938) and Wright (1977). Much like discriminating colours by their wavelengths, unique scents are attributed to a unique spectrum of signals: the combinatorial code. Unfortunately, the case of mirror image molecules refutes this theory: these molecules would exhibit exactly the same spectra given their symmetry. Furthermore, even if the discrimination of smell was purely vibrational, how would this signal be measured? Like shape, how does the receptor detect the different vibrations between different molecules?

**Figure 2.**
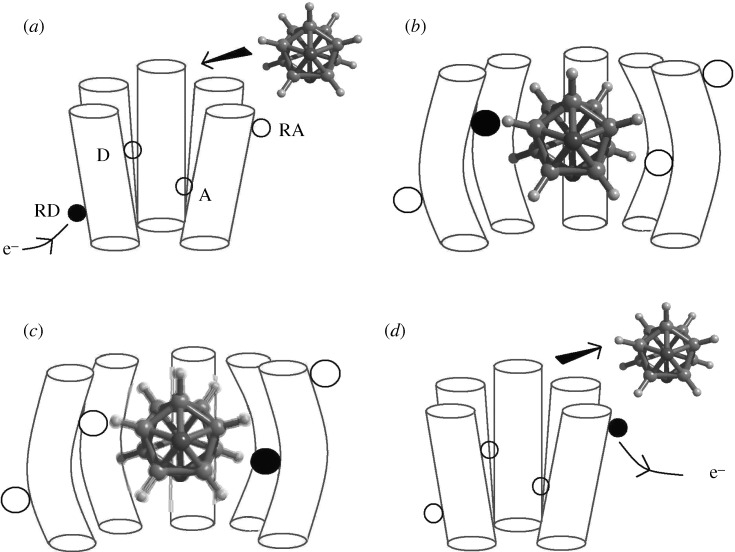
The proposed sequence of events according to Turin’s theory of signal transduction is shown. The olfactory receptor is pictured here as a cartoon with five cylinders to represent the protein helices (there are typically seven); the odorant is a carborane isomer—a camphoraceous smelling molecule (Turin & Yoshii 2003). (*a*) Source of electrons available at RD. (*b*) Electron tunnels to site D (donor) as odorant docks and deforms receptor. (*c*) Electron tunnels to A (acceptor) mediated by odorant phonon. (*d*) Odorant is expelled and electron transmission to RA initiates signal.

Turin (1996) proposed a theory to include quantum mechanics in our understanding of smell. He postulated that the olfactory receptor contains electron donor (D) and electron acceptor (A) units which are separated in energy by a fixed amount that matches a specific odorant’s quanta of vibration (a phonon). Upon an odorant binding to its receptor, an electron tunnelling event occurs when the emitted phonon fills this D–A gap, which in turn may initiate the transmission step towards the brain. Once the electron reaches A, the signal is initiated via the G-protein release mechanism. The D and A are electron sources and sinks, respectively, as part of the receptor protein which provides the tunnelling electron which is the message carrier (figure 2).

Neither vibration-based nor shape-based theories of olfaction have fully satisfied scientific scrutiny to date. However, the introduction of quantum mechanics into describing the initial processes of olfaction can no longer be overlooked as it provides physical validification of Turin’s original idea. This model of olfaction incorporating quantum mechanical formalism manages to detail a receptor’s odorant discrimination based on simple oscillations while successfully distinguishing between mirror images of a molecule without violating any fundamental physical rules (Brookes *et al.* 2007). This model, and the application of the ‘swipe card’ paradigm to cover generalizations of models like Turin’s, is described below.

## 3. A swipe card model

### (a) Good, good, good, good vibrations

Vibrations are everywhere; from the quartz crystal that times the hands on your watch to the spring in a kangaroo’s hop. These examples are simple harmonic oscillators (SHOs). The bonds in a molecule, such as an odorant molecule, can also be approximated as SHOs. The nuclei of a molecule with mass *m*, displaced at small distances from an equilibrium position, tend back to their starting points under the forces of the surrounding electrons. These motions are restorative in a way described by Hooke’s Law *F*=−*kx*, the ‘force is as the extension’. By integration of this force the potential energy can be determined, 
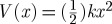
. The first derivative, where *F*=−∂*V* /∂*x*=0, determines the equilibrium position—the most relaxed state with least energy, the state that most of nature wishes to be. Plotting the potential energy *V* (*x*) versus displacement *x* provides a parabola from which the motion is characterized, and the *spring constant* *k* can be found. Thus, simple harmonic motion can be characterized by a simple curve. Using Hooke’s Law and solving the *time-independent Schrödinger equation* for quantum behaviour, solutions show the atomic modes of motion are quantized by 
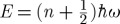
, where 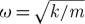 (its angular frequency) and *n* indicates the quantum level (the level of phonon excitation). At the simplest level of approximation, and under the application of a coherent driving force, these modes of motion, *normal modes*, are molecular dances the atoms make of concerted motion whereby every atom passes through its equilibrium position at the same time. The centre of mass of the molecule never changes and each mode is independent (never exchanging energy with another mode). Though molecular vibrational spectra is not entirely harmonic (there are higher order terms), at small displacements from the equilibrium, we can make a useful approximation that each mode of vibration is like a simple harmonic oscillator.

### (b) Can quantum mechanics explain how humans smell?

In Turin’s theory, signal transduction is determined by an electron *transition* from donor (D) to acceptor (A). Quantum transitions such as these between quantized electronic and vibrational states in atomic levels are reliably and accurately calculated using *Fermi’s golden rule*. The golden rule determines a probability per unit time that a transition between two *zero-order states* occurs under the presence of a small perturbation. It is appropriate to apply this rule to Turin’s theory of olfaction in order to determine whether the electron may cross from the D and A state under an influencing force, perturbation, of an odorant. Quantum mechanically speaking, there is a finite possibility that the tunnelling electron may be anywhere. Therefore, it needs to be determined whether this possibility of the electron getting from D to A is more favourable in the presence of a ‘correct’ odorant than when there is either no odorant or an incorrect odorant coupled with a particular receptor.

**Figure 3.**
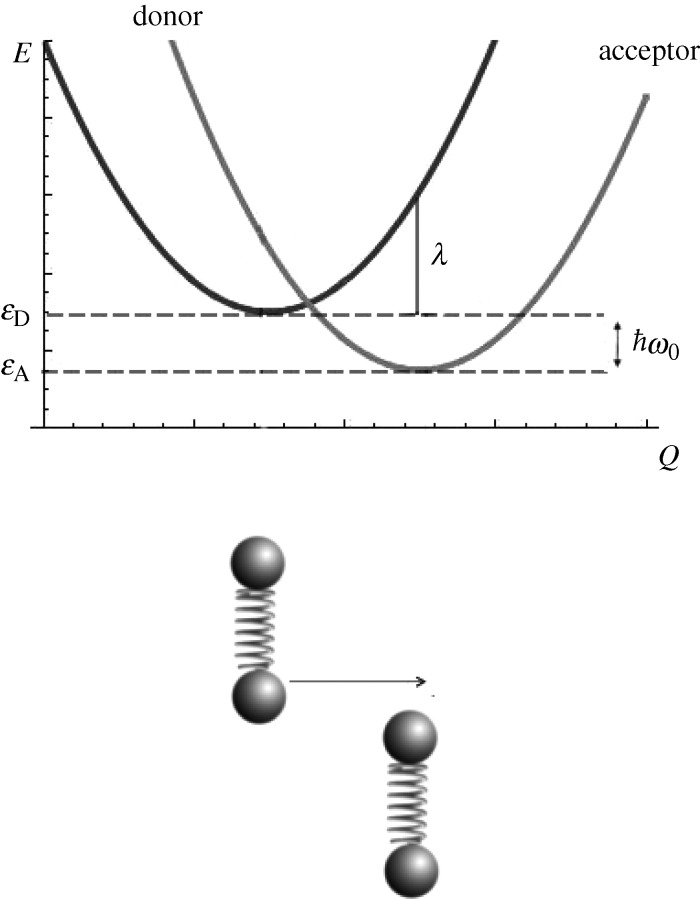
The *configuration coordinate diagram* to describe events in olfaction is shown. Electron tunnelling from the donor |*D*〉 to acceptor |*A*〉 is facilitated by the excitation of an appropriate odorant phonon corresponding to 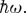 The change in force as the electron transfers is characterized by the shift in energy, down the vertical axes *E*, and displacement, along the reaction coordinate *Q*, that is phonon assisted. The reaction coordinate describes the displacements of nuclear modes that entail the reaction pathway.

A *configuration coordinate diagram* helps us to put the electron transition in the context of nuclear vibrations. The coordinate diagram uses two parabolas (figure 3) to describe the harmonic motions of all oscillations within the receptor, initially (in state D) and finally (in state A). This approximates all the SHOs as one collective motion. The nuclear modes of motion, not necessarily the *normal* modes, that describe the reaction pathway (electron on D or A) consist of the reaction coordinate. There are two instances (channels) when an electron can transfer from D to A while satisfying the fundamental law of energy conservation: when 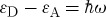 or when *ε*_D_−*ε*_A_=0. The first instance corresponds to receptor discrimination of an odorant and the second does not. In the non-discriminating channel the odorant is not excited. In the discriminating channel, the odorant absorbs this energy 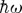. The probability for both events, discriminatory and non-discriminatory, can be calculated with Fermi’s golden rule. For quantum mechanics to explain how humans smell, the discriminatory channel must ‘win’.

The presence of an odorant introduces a *non-adiabatic* interaction between donor and acceptor whereby an electron may make a quantum jump from one energy state to another: from |*D*〉 to |*A*〉. We assume that these states are sharp electronic energy levels that couple only weakly with nuclear transitions. Any strong interaction would widen lifetime broadening and obscure selectivity. In one way the presence of an odorant introduces an electronic state like a ‘stepping stone’ for an electron to hop from the electronic states D to A. The strength of this hopping is determined by the electronic part of a *transition matrix element*. This is the electronic contribution to the non-adiabatic component which determines the ease with which the electron can transfer across these states.

As the electron transfers from D to A the odorant feels a change in force which springs the key normal mode into action (excites the relevant odorant vibration). This change in force is measured by the electron–phonon coupling, a *Huang–Rhys factor* (*S*). *S* is a ratio of the relaxation energy *λ* (or reorganization energy) to the phonon’s energy 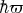. The relaxation energy is determined from the change in force incurred as the electron moves from D to A in the field of the oscillating odorant. Thus, those IR active phonons, where there is a change in dipole moment, are those detected in this model. All other vibrations in the protein are either too low in frequency or too far away from the binding sites to contribute to the *S* of the odorant and interfere with recognition (Brookes *et al.* 2007).

By analysis, the crucial result given by the application of the golden rule is that the discriminating case has nearly a 600× higher rate of transmission than that of the non-discriminating case. This holds true when: we use harmonic approximations, background oscillations are low frequency and weakly coupled to the electron transfer, there is a low reorganization energy and when one phonon of vibration in the odorant is excited. Under these conditions the inelastic channel is preferred and the odorant with the ‘right’ frequency will be detected over the ‘wrong’ one owing to resonance transfer of bond vibrations communicated between the odorant and receptor. This provides a model that defines the gate-keeping nature of olfactory receptors.

### (c) A ‘swipe card’ model

For a fast and discriminating rate of electron transfer, a strong mixing of the right electronic and vibrational states is required. Both states are affected by the *structure of the molecule*. A compromise of geometrical (shape) factors is required in combination with the right energetic (vibrational) factors. This model, therefore, incorporates principles from the lock and key model whereby the shape of an odorant is important. However, this differs from the lock and key model because it describes the next step in signal transduction, which the lock and key does not. This model may be considered much like a ‘swipe card’ (or a key card) where an approximate fit between odorant and receptor (shape) is necessary to swipe the key into the lock, but it is the internal message (vibration) that is essential to open the door. Thus, shape is necessary but not sufficient.

### (d) Some answers, and more questions

How, then, can we quantify smell? The swipe card model *tests* the physical feasibility of Turin’s postulate and finds that a smell signal *can* be quantified by the rate of electron transfer. Further the odorant combinatorial code may be calculated by measuring the electron–phonon coupling (Huang–Rhys factors) for each odorant mode and plotting an odorant characterizing spectrum. The change in force owing to electron transfer is sensitive to the direction of the oscillating charges in the odorant, and this is calculated in the Huang–Rhys factor. Mirror image molecules, while they will have identical 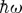, will have differing Huang–Rhys factors owing to the contrast in the directions of the relevant oscillating atoms, when they are held in the same chiral (and thus symmetry breaking) receptor. The odorant combinatorial code has to differ by the activation of only one extra receptor type to drastically redefine a smell. In (*4R*)-carvone, for example, it may be that the directions of the oscillating atoms maximize the change of force that is measured in the electron–phonon coupling. So the swipe card model even explains the apparent mirror image molecule oddities. Though still untested, the swipe card at the very least provides explanation and a method of prediction as to how the olfactory receptor gate keepers may respond to certain odorants. Questions that face scientists in olfaction now must include those that really challenge and test the theory of smellable vibrations. Can humans discriminate isotopes at the receptor stage? Can electron transfer at the receptor be detected by experiment? How well does the Huang–Rhys factor, and the odorant spectrum, define and predict other oddities in smell? What can knowing more about signal transduction in olfaction tell us about generic signal transduction mechanisms?

## 4. The future

**Figure 4.**
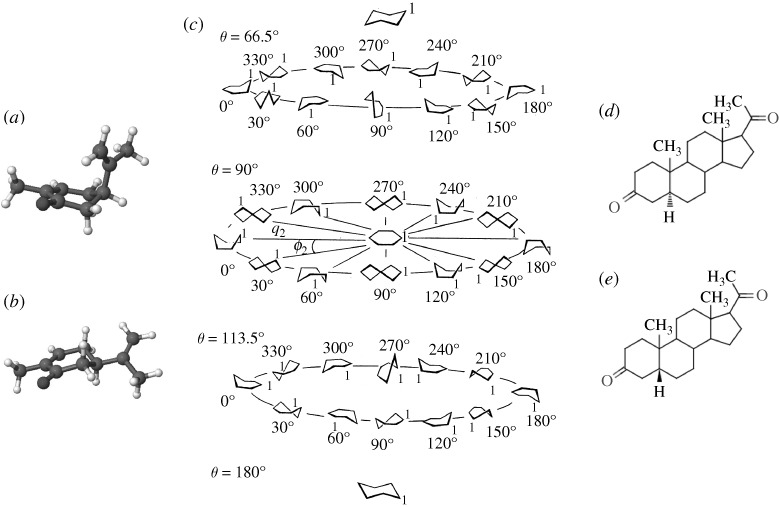
(*a*) (*4R*)-(−)-carvone with the isopropenyl group axial to the ring. (*b*) (*4R*)-(−)-carvone with the isopropenyl group equatorial to the ring; adapted from Brookes *et al.* (2009). (*c*) The ‘twist’ , ‘boat’ and ‘chair’ states (and deviations inbetween) in cyclohexane; adapted from Juaristi (1995). Also, the difference (or lack of) between two-dimensional structures of (*d*) 5α-*diH*- and (*e*) 5β-*diH*-progesterone is shown.

Mirror image molecules are of interest because they exemplify the importance of shape in receptor detection, while still leaving the rules of shape selectivity obscure. They clearly show that the positions of atoms matter, though we still lack a scientific explanation as to why. A recent study by Brookes *et al.* (2009), which categorizes a suite of mirror image molecules documented by Leffingwell and associates, finds that, by categorizing the odorants by their scent descriptors and physical attributes, a simple rule can be determined. The rule is that odorant molecules of an enantiomer pair will smell alike (type 1) when they are rigid, and will smell different (type 2) when they are flexible (Brookes *et al.* 2009). This study of flexibility determined that those odorant molecules containing six-membered rings can twist and pseudo-rotate between ‘twist’-, ‘boat’- and ‘chair’-like configurations, similar to the flexibility seen in cylcohexane (figure 4), or have *cis–trans* stereo-isomeric flexibilities. This begs the question: which structure is it that is recognized by the receptor? It is perhaps more relevant to ask which shape turns the receptor ‘on’ as opposed to which shape allows the odorant (ligand) to get there. Note that the degree of flexibility will affect recognition at the site (affinity) but also the signalling or switching (efficacy/actuation). I propose that the evidence of differentiable mirror image odorants determines the importance of flexibility in olfactory actuation. It is common in the relevant literature these days to propose that flexibility aids the affinity a ligand has for a receptor site. This would imply, however, that two mirror image related molecules, equal in degrees of flexibility, would activate an equal set of receptors and smell the same, when they often do not. The inference that greater flexibility determines a more promiscuous ligand is not valid here. Flexibility could be as much a hindrance as an aid when it comes to ligand–receptor actuation.

Odorants are not the only small molecules that interact unpredictably with large proteins; steroid hormones, anaesthetics and neurotransmitters, to name a few, are examples of ligands that interact specifically with special receptors to produce important biological processes. Steroids, in particular, exhibit similar curiosities to odorants. Compare 5α-*diH*- and 5β-*diH*-progesterone, for example, in figure 4. The only difference structurally between these ligands is the direction of one carbon–hydrogen σ-bond. Two dimensionally, the differences in structure are almost undetectable, yet the effect *in vivo* is quite drastic whereby the two steroids produce different bio-effects (Galigniana *et al.* 2004). It cannot be clearer that minute changes in a ligand’s structure can have a profound impact on activity. This is exemplified not only by mirror image odorants, as discussed above, but also in the endocrine system, where even smaller stereo-isomeric changes to a molecule make a difference to the molecule’s function.

Research thus far shows that models based on shape or vibrations alone are not enough to describe and predict ligand performance. The ‘swipe card’ model combines these physical attributes, and does better. This electron transfer model depends intimately on a Huang–Rhys factor, which in turn depends on the orientation of the critical oscillating mode of vibration. Where conventional inelastic electron tunnelling spectroscopy can determine the orientation of a molecule (Kirtley *et al.* 1976), so could olfactory-based inelastic electron tunnelling. It is interesting to hypothesize that, given the positions of atoms can certainly be detected by the electron, perhaps any flexibility in the odorant may promote or demote the electron transfer in an actuating step. Thus, we can make testable conjectures and attempt to characterize scent. Also important is that this simple ligand-perspective analysis has shown that it is pressing to consider the physics of a dynamical quantum world, where minute changes have gargantuan effects, as opposed to the useful but static textbook ball and stick models.

## 5. Discussion and conclusions

What we smell is very curious. When Alice in wonderland ponders at her reflected world ‘I wonder if looking glass milk is as good to drink’, she could have been thinking of mirror image molecules: many odorants related by symmetry *do not smell the same* (Leffingwell and associates). Some odorants change in character with concentration; for example, *p*-meth-1-en-8-thiol, which turns from grapefruit abruptly to sulphurous (Wilson & Stevenson 2006). Some odorants smell sulphurous even when they do not contain sulphur (Turin 1996). Some odorants may share the same atoms in the same order, and differ only in the direction of one bond. Whether one hydrogen atom is axial or equatorial to the plane of the rest of the molecule, which in turn induces dramatic changes to the other atoms geometrically, may drastically alter the smell of the odorant. Some odorants are very subtle, some are very strong and some conjure vivid memories in ways our other senses cannot. Perhaps it is curiosities such as these that cause a certain scepticism of smell: demonstrated by an attitude that it is ‘all in the brain’. Let it be emphasized here that there certainly is a difficult explanation of the way certain people interpret smell. However, this is true of all perception and feeling. What the brain *does* with the information obtained may vary from person to person, but, as in sight and hearing, there must be a common way we achieve the information in the first instance. While the neuroscience and psychology surrounding this area is doubtless interesting, the scope of this article examines the *initial* processes with the outside world. Leaving the piquant question—just what are the distinguishing characteristics of smell? A scientist can determine a colour by measuring the wavelength of the responsible photons. A clothing chain store can match exactly the colours of the suit jacket bought in a London store with the trousers that may have been bought in Edinburgh, and this is done using 16 wavelengths. For smell, there may be many more, adding to the complications scientists already face, but that does not mean it is impossible to match smells. It is put here that the scientist may be able to measure smell by determining the relevant odorant oscillation. Though it is essential that more conjectures and refutations are made to justify this claim and establish the validity of the swipe card theory, at least now in olfactory science the right questions are beginning to be asked. It is the question of how the odorant communicates with the receptor (and in general the ligand and the protein) that concerns this article, and a question which begins an interesting era of scientific discovery.

## Author Profile

**Jennifer C. Brookes**

**Figure:**
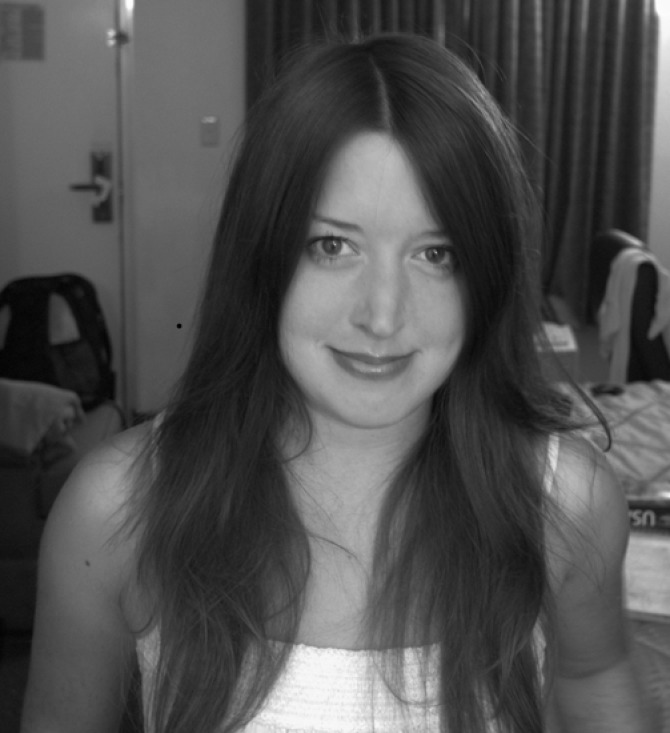


From 2001 to 2004, Jennifer Brookes read joint honours physics and philosophy, at King’s College London (KCL). In the summer of 2003 she held a short studentship at KCL in a laboratory under the supervision of Gordon Davies, examining in silicon the defects, interstitials, substitutionals, doping and impurities that can be exposed using infra-red absorption spectroscopy. For her undergraduate work she won the Perkin Elmer prize for practical physics. Wanting to learn particular theoretical techniques she began in 2004 an MSc in physics, at University College London (UCL), for which she won a Graduate School Masters Award to fund the ambition. It was then, as part of a research project, that she was introduced to future PhD supervisors Andrew Horsfield and Marshall Stoneham and the imaginative ideas of Luca Turin’s theory on olfaction. Jennifer began her PhD studies at UCL in 2005, thrilled to be given the opportunity to investigate the physics of this intriguing sense with these scientists. In December 2008 Jennifer successfully defended her PhD thesis and won a departmental prize for Outstanding Postgraduate Research in Condensed Matter and Materials Physics. In 2009, she worked with Marshall Stoneham, as research assistant on various projects at the London Centre for Nanotechnology at UCL. These projects began to be of interest in olfaction, but have shown interesting insights into problem solving in other signal transduction events. In particular, her recent work, in collaboration with Gavin Vinson, of Queen Mary, University of London, finds interesting correlations of ligand dynamics with bio-function *in vivo*. In June 2009 Jennifer was awarded a Sir Henry Wellcome post-doctoral fellowship that gives her a great opportunity to pursue her interests in olfaction and a wider remit of ligand (drug)–protein (receptor) interactions. Her fellowship will be hosted by UCL for the 4 years duration, half of which she shall spend as a visiting fellow at the Massachusetts Institute of Technology (MIT) where she will work in Shuguang Zhang’s laboratory at the Center for Biomedical Engineering. In her spare time she loves to travel, dance and swim.

## Footnotes

1 That is, with many more degrees of freedom.
